# Curative resection of a primarily unresectable acinar cell carcinoma of the pancreas after chemotherapy

**DOI:** 10.1186/1477-7819-7-22

**Published:** 2009-02-25

**Authors:** Marius Distler, Felix Rückert, Dag D Dittert, Christian Stroszczynski, Frank Dobrowolski, Stephan Kersting, Robert Grützmann

**Affiliations:** 1 Department of General-, Thoracic- and Vascular Surgery, University of Dresden, Dresden, Germany; 2 Department of Pathology, University of Dresden, Dresden, Germany; 3 Department of Radiology, University of Dresden, Dresden, Germany

## Abstract

**Background:**

Acinar cell carcinoma (ACC) represents only 1–2% of pancreatic cancers and is a very rare malignancy. At the time of diagnosis only 50% of the tumors appear to be resectable. Reliable data for an effective adjuvant or neoadjuvant treatment are not available.

**Case presentation:**

A 65-year old male presented with obstructive jaundice and non-specific upper abdominal pain. MRI-imaging showed a tumor within the head of the pancreas concomitant with Serum-Lipase and CA19-9. During ERCP, a stent was placed. Endosonographic fine needle biopsy confirmed an acinar cell carcinoma. Laparotomy presented an locally advanced tumor with venous infiltration that was consequently deemed unresectable. The patient was treated with five cycles of 5-FU monotherapy with palliative intention. Chemotherapy was well tolerated, and no severe complications were observed. Twelve months later, the patient was in stable condition, and CT-scanning showed an obvious reduction in the size of the tumor. During further operative exploration, a PPPD with resection of the portal vein was performed. Histopathological examination gave evidence of a diffuse necrotic ACC-tumor, all resection margins were found to be negative. Eighteen months later, the patient showed no signs of recurrent disease.

**Conclusion:**

ACC responded well to 5-FU monochemotherapy. Therefore, neoadjuvant chemotherapy could be an option to reduce a primarily unresectable ACC to a point where curative resection can be achieved.

## Background

Acinar cell carcinoma (ACC) of the pancreas is a very rare tumor entity, since only 1% of the exocrine pancreatic malignancies derive from acinar cells [1]. These carcinomas mainly occur between the 5^th ^and 7^th ^decades of life. Symptoms like weight loss, abdominal pain, nausea and vomiting are non-specific and mostly related to either locally advanced tumors or metastasis [1]. Some patients show fat necrosis, polyarthralgia and eosinophilia due to increased serum-lipase levels (lipase hypersecretion syndrome) [1-3].

In general, half of the patients have advanced disease with either metastasis or a locally unresectable tumor at the time of diagnosis. Median overall survival at this stage is about 19 months, and has been reported to be between ductal adenocarcinoma of the pancreas (median survival nine months) and pancreatic neuroendocrine neoplasma (median survival 40–60 months) [2]. After tumor resection, patients with ACC show a better prognosis, up to a median survival time of 36–41 months [4]. Today, surgical therapy is the only curative approach, although ACC has a high recurrence rate of 72% after this treatment [2].

Up to now, ACC of the pancreas has been described by few case reports [1,5-7]. Only one clinical study presented a larger number of ACCs [4]. No prospective clinical trials are available, and adequate treatment for the advanced disease remains a topic for discussion. However, a report from Lee et al. [6] showed a significant tumor reduction under treatment with capecitabine and concurrent radiotherapy. Other authors report that ACC tumors responded well to intra-arterial combination chemotherapy consisting of 5-FU, mitomycin and cisplatin [2,8].

We report for the first time a partial tumor remission under mono-chemotherapy with 5-FU and subsequent potential curative resection of a primarily unresectable ACC.

## Case presentation

A 65-year old male with obstructive jaundice (bilirubin 126 μmol/l) and non-specific upper abdominal pain was admitted to the hospital in December 2005. Laboratory parameters showed pathological values: ALAT/ASAT 10.2/4.5 μmol/l (< 0.85/< 0.85 μmol/l), YGT 23.1 μmol/l (< 1.19 μmol/l), amylase/lipase 5.1/23.2 μmol/l (0.22–0.88/< 1.00 μmol/l) with a mild elevation of the inflammatory parameters (leukocytes 13.6 gpt/l and CRP 22 mg/l), and CA 19-9 was elevated to 968.8 U/ml. The tumor markers CEA and AFP were within the normal ranges. There were no signs of subcutaneous fat necrosis or arthralgia.

**Figure 1:**
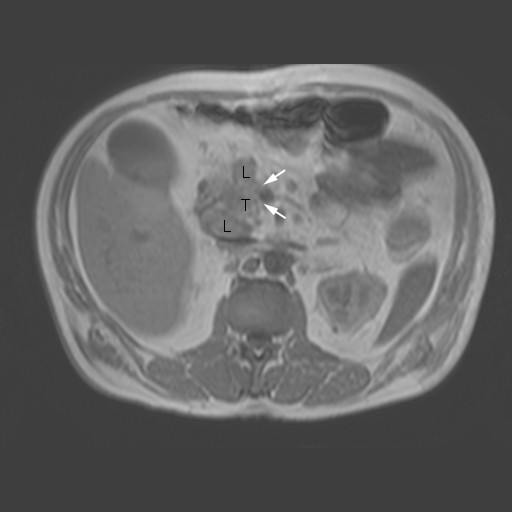
**T1 weighted MRI at time of diagnosis**. T = tumor; L = lymph node; arrows indicating semicircular contact to the superior mesenteric vein.

**Figure 2:**
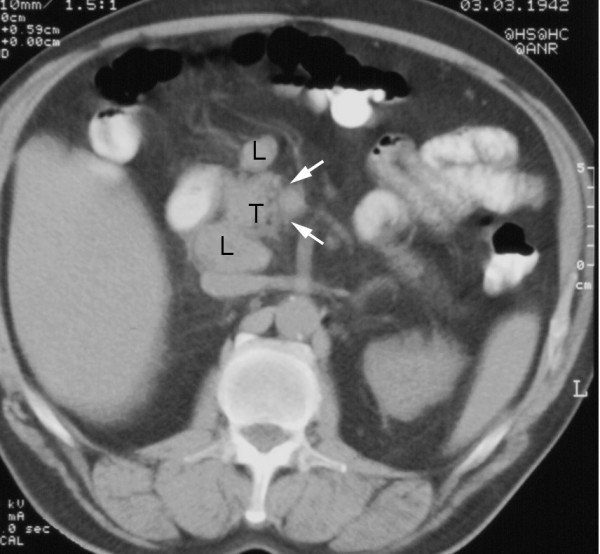
**CT at time of diagnosis before chemotherapy**. T = tumor; L = lymph node; arrows indicating semicircular contact to the superior mesenteric vein.

On MRI T1 weighted images, an isointense tumor in the pancreatic head was visible. The axial diameter of the tumor was about 4 cm; it contacted the portal vein and the superior mesenteric vein and it seemed to infiltrate the hepatoduodenal ligament. Two enlarged lymph nodes were noted at the upper and lower margin of the pancreatic head (Figure 1). There was no proof of metastatic spread. The CT scan before chemotherapy confirmed these findings (Figure 2). Differentiation between inflammation and malignancy and any prediction concerning curative resection were not possible at that time. ERCP showed the typical double duct sign, and a plastic stent was placed during this procedure. Endosonography confirmed the tumor in this location, and a fine needle aspiration biopsy was taken simultaneously.

**Figure 3:**
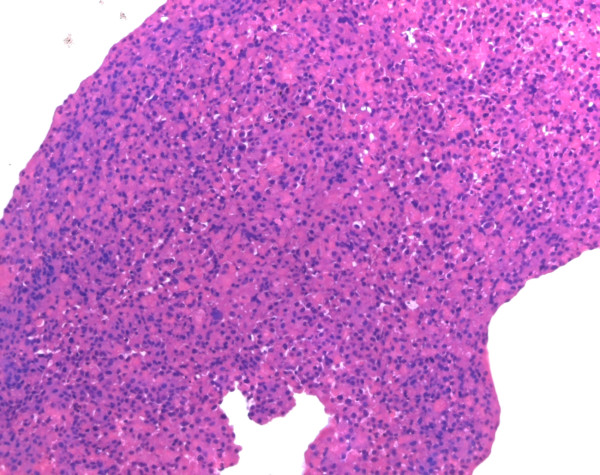
**Acinar cell carcinoma: solid sheets of eosinophilic tumor cells with dark round nuclei**. Preoperative H&E morphology, original magnification ×25.

In the histology of the tissue, the normal acinic structure of the exocrine pancreas was effaced. The irregularly shaped tumor cells with eosinophilic cytoplasm had rounded dark nuclei and were growing in solid sheets. No ductal structures could be observed in the tumor tissue (Figure 3). Markers of endocrine differentiation were negative; only immunohistochemistry indicated a tumor of the exocrine pancreas. Lastly, acinar cell carcinoma was confirmed by immunoreactivity of the tumor to trypsin.

**Figure 4:**
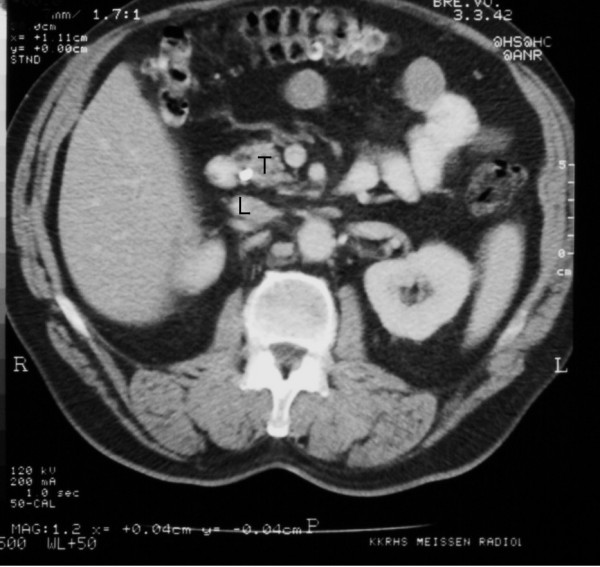
**CT after 5 cycles of neoadjuvant chemotherapy with 5-FU**. T = tumor; L = residual precaval lymph node.

After stenting, the patient showed a total decline in jaundice and, under analgetics, no abdominal pain. Therefore, surgical exploration with the aim of total tumor extirpation was considered. Bulky tumor masses were found to infiltrate the hepatoduodenal ligament, the interaortocaval space and the venous confluens of the superior mesenteric and the portal vein. The ACC was therefore determined not to be resectable. A fine needle aspiration biopsy was taken from the pancreatic head, and the operation was completed as an explorative laparotomy. Again, the diagnosis of an ACC was reconfirmed by histopathology. After implantation of a venous port system, palliative chemotherapy was given to the patient. Altogether, the patient was treated with 5 cycles of 5-FU monotherapy (220 mg/m^2^/d); treatment occurred over eight weeks with a break of two weeks per cycle. There were no major complications during the chemotherapy. Only one time the patient showed mild diarrhea and a common cold. An uropathy was treated with systemic antibiotics. Overall, the patient stayed in good general condition. The biliary stent was changed twice without any complications. CA 19-9 was determined as a tumor marker after each chemotherapy-cycle. After the second cycle, CA 19-9 already showed a normal value of 11.0 Units/ml; after one year, CA 19-9 was 18.6 Units/ml. After five cycles of treatment (one year), a CT for restaging was performed again. The images showed an obvious reduction in the size of the tumor. The slightly hyperintense area in the head of the pancreas had diameters of barely 2.4 × 1.1 cm, with a small (1.0 cm) residual precaval lymph node (Figure 4). There had been no other suspicious lesions, especially in the liver or the peritoneal cavity.

At this stage, the patient was sent to our high volume specialized pancreatic cancer center. During further endosonographic examinations, the small tumor size (1.4 × 1.8 cm) in the pancreatic head was confirmed. Transcutaneous abdominal ultrasound showed no signs of unresectability or metastatic spread. The PET scan indicated no vital tumor tissue in the pancreatic head.

We decided upon operative reexploration with the aim of total tumor resection. A PPPD without any intraoperative complications was performed; because of adhesions at the portal vein, a partial resection (3 cm) of the vessel had to be carried out simultaneously. All intraoperative histopathological examinations of the resection margins were without any tumor tissue. The postoperative hospitalization stay proceeded regularly and without any adverse events.

**Figure 5:**
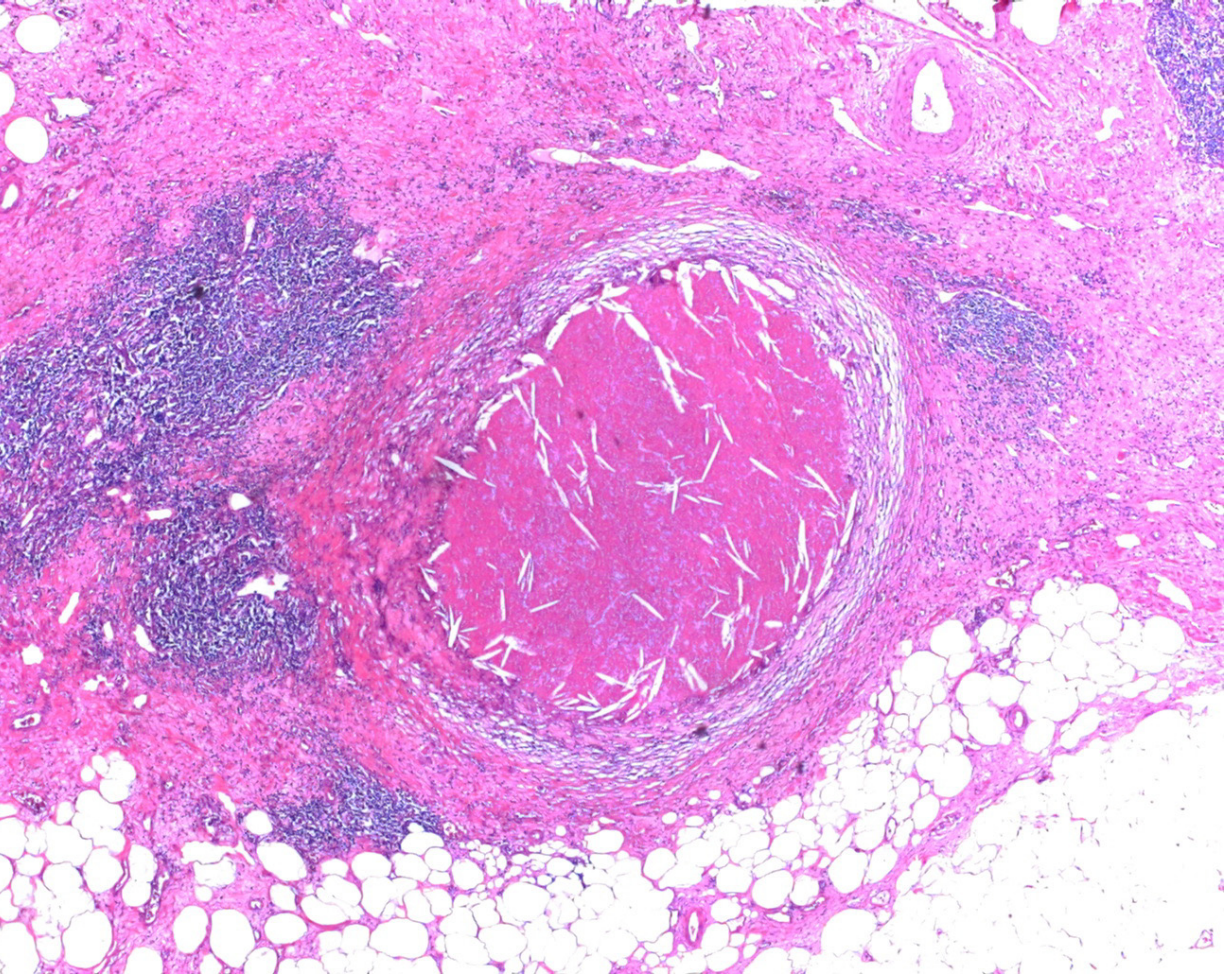
**Pancreatic and peripancreatic tissue with an area of necrosis after neoadjuvant chemotherapy**. H&E morphology, original magnification ×25.

**Figure 6:**
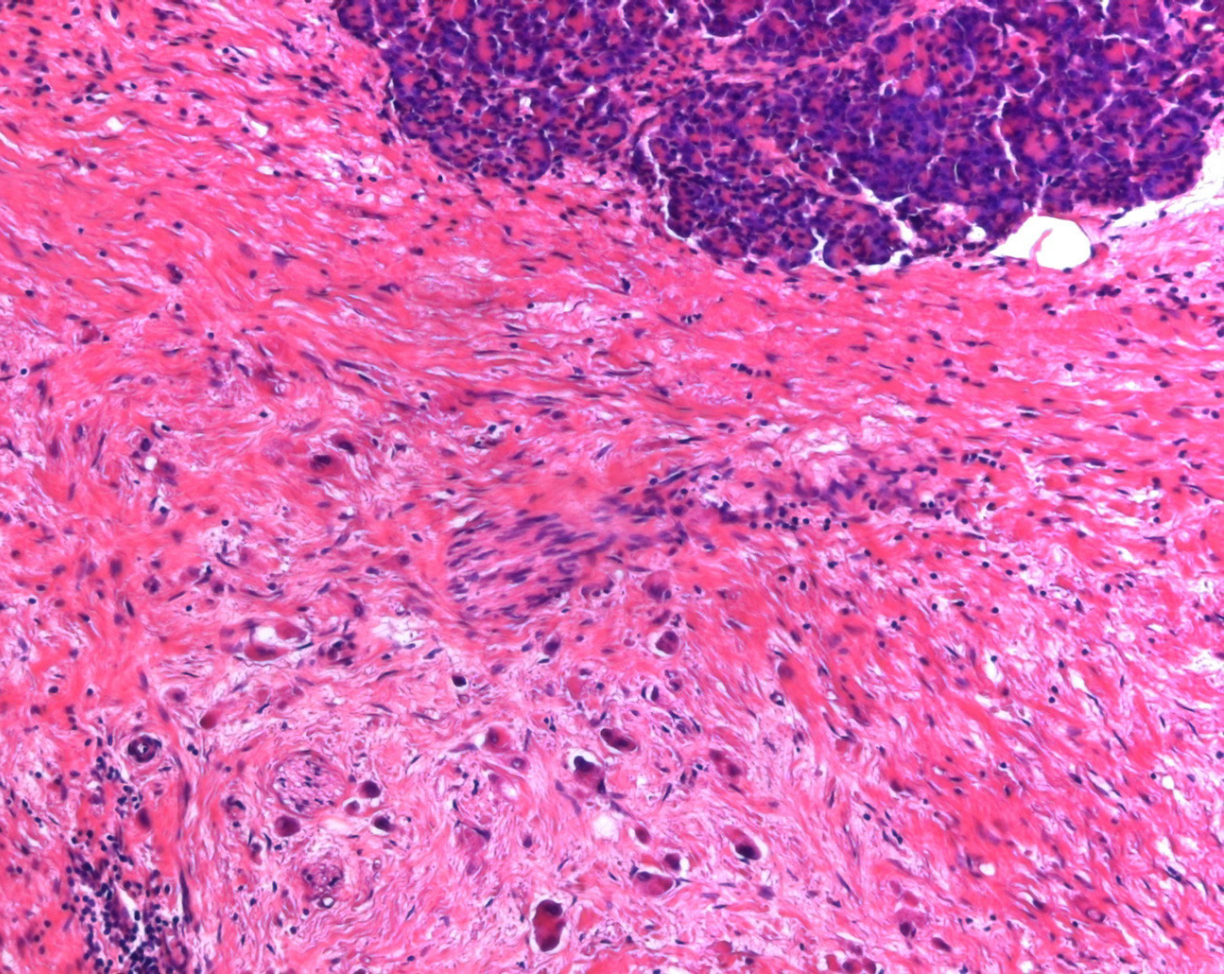
**Pancreatic tissue with normal acinar lobules at the top surrounded by dense fibrotic tissue that contains scattered irregularly shaped large tumor cells in the lower half of the picture**. H&E morphology, original magnification ×100.

The histopathological examination of the resected tumor tissue showed areas of necrosis surrounded by fibrotic stroma with a folliculoid lymphatic reaction (Figure 5). In between resting exocrine lobules, scattered pleomorphic tumor cells and small groups of tumor remnants (up to 2 mm in diameter) were detected (Figure 6). Only a few vital tumor cells were located in the peripancreatic fat. Metastatic tumor was found in two of nine regional lymph nodes, and all margins were tumor-free, resulting in a postoperative tumor classification of ypT3, ypN1 (2/9), ypMx R0 according to UICC 2003.

Any further adjuvant therapy was refused by the patient. Regular checkups were done in our pancreatic clinic. As of now, about 18 months after operation, the patient shows no clinical symptoms or ultrasonographic or laboratory signs of recurrent disease. CA 19-9 is 8.1 U/ml.

## Discussion

Acinar cell carcinoma of the pancreas is a very rare tumor entity, and in the literature there are only small retrospective studies and a few case reports on this matter [1,2,4,9-11]. In our high volume pancreatic center, with up to 100 pancreas resections per year, we have seen only three cases of ACC in about 14 years. Klimstra et al. [1] and Holen et al. [2] reported a median age of about 60 years for ACC onset. At the time of diagnosis, our patient was 65 years old and showed obstructive jaundice as a consequence of the pancreatic head tumor. As reported by Klimstra et al. [1] obstructive jaundice seems to be infrequent with ACC (12%) because the tumor is less often located in the head of the pancreas than ductal-adenocarcinoma (53%) [2].

Commonly referred clinical symptoms are upper abdominal pain, weight loss and nausea/vomiting [1,2]. In our case, the patient had non-specific abdominal pain and jaundice. At time of diagnosis our patient showed, in addition to the elevated jaundice parameters, slightly elevated serum-amylase (5.1 μmol/l) and high lipase (23.1 μmol/l) levels in combination with a mild elevation of the inflammatory parameters (leukocytes 13 Gpt/l, CRP 22 mg/l). However, there were no symptoms correlated with the formerly described lipase-hypersecretion-syndrome [3]. This corresponds to the literature, which identified lipase-hypersecretion-syndrome as a very rare finding in ACC patients [1,2,12]. It is important to mention that with the constellation of laboratory parameters and clinical signs described, even acute pancreatitis has to be considered.

The initial elevated tumor marker CA 19-9 (968.8 Units/ml) was used to monitor the efficiency of the therapy, especially because we could not detect any elevation of other tumor markers, e.g., AFP, which were mentioned as possible markers of acinar cell differentiation within these tumors [11,13,14].

Tatli et al. [15] described ACCs as usually exophytic, oval or round, well marginated and hypovascular masses on CT or MRI scans. In the initial MRI, our patient showed a contrast-enhancing solid mass, which is a typical presentation for a small ACC, as described by these authors. However, in contrast to Tatli et al. [15], our tumor was not well marginated and was rather indefinable from the pancreatic tissue.

The diagnosis of an ACC cannot be made with a high reliability unless histopathological examination has been performed [12]. But in cases with suspicion of pancreatic cancer, percutaneous thin-needle biopsy should not be performed because of possible tumor cell dissemination [16]. Therefore, explorative laparotomy with or without resection is necessary if there is any indication of malignancy.

Endocrine and acinar tumors, as well as even rarer pancreatoblastoma, normally lead to few formation of fibrosis and thus are often cell-rich tumors with cells not arranged orderly and rounded inconspicuous nuclei. Differentiation between endocrine and acinic tissue can be achieved by immunohistology, because endocrine tissue will react with antibodies against synaptophysin or chromogranin. Antibodies against trypsin or lipase can be used to prove acinic differentiation, but, as ACC are scarce, only a few centers have these antibodies in stock or are accustomed to using it [1,17]. With a set of endocrine and exocrine markers, the rare cases of mixed acinar-endocrine carcinomas can be separated, if either differentiation makes up for at least 25% of the tumor. Exceedingly rare are tumors which contain neoplastic ductal structures as well and can be highlighted by expression of CK7 or mucin 1 [18].

Reviewing the literature, operative resection of the tumor seems to be the only curative treatment of ACC [1,2,4,12]. Therefore, if an ACC is assumed or histologically proven, the chances for total resection should be surveyed intensively. Kitagami et al. [4] reported a 5-year survival rate of 43.9% in the case of total resection (without resection: 5-year survival rate 0%) [4]. Holen et al. [2] showed a median actuarial survival rate of 36 months for those patients initially treated by operative resection.

However our knowledge of the efficiency of any neoadjuvant or adjuvant treatment with radiation or chemotherapy for ACCs is limited. Only in small studies or case-reports are the effects in patients with mostly unresectable ACCs described [6,7,12]. For the first time, Seth et al. [12] reported on four cases with successful neoadjuvant chemoradiation, which made total resection of the tumor possible. In this context, agents like 5-FU, gemcitabine, cisplatin and adriamycin are mentioned [12]. Other authors [7] observed an increase of survival time by using several combination regimes with gemcitabine, irinotecan, oxaliplatin, docetaxel capecitabine, 5 FU, leucovorin, erlotinib, sunitimib and sirolimus. Lee et al. [6] reported on two cases in reducing the tumor size by capecitabine and concurrent radiotherapy. In summary, there are no evidence-based procedures for neoadjuvant or adjuvant treatment, mainly because of the low incidence of ACCs.

However, our case is unique and of great importance, since never before has any reduction of the tumor size (from 4 cm to 2.4 cm) under neoadjuvant mono-chemotherapy with 5-FU been observed, followed by the curative resection of an ACC. Comparison of the histology of the biopsy and the surgical specimen showed large differences, because after chemotherapy there were necrotic areas, and the vital tumor tissue was much reduced. Highly cellular tumor tissue of relatively uniform nuclei had vanished, and instead, little groups and scattered single tumor cells were fenced in by dense fibrosis, and cellular pleomorphism indicated degenerative changes after chemotherapy.

As mentioned above, we used the chemotherapeutical agent 5-floururacil. 5-FU is commonly used for adjuvant treatment of pancreatic ductal adenocarcinoma. The ESPAC-1 trial especially showed significantly positive impact on long-term survival in patients with PDA [19].

In the present case, after neoadjuvant treatment with 5-FU only minimal tumor tissue remained in situ, and it could be removed completely with negative margins (R0) by surgery. Therefore, 5-FU seems to have a significant effect on ACC tumors, as shown in our patient. However, any final statement is not possible yet, as it has only been 18 months since surgery.

Considering our case, it is important that any patient with an ACC be restaged, and, if possible, re-explored in a specialized high volume center in particular, because surgical resection seems to be the only curative therapy. If the ACC is unresectable, the patient should be treated by 5-FU chemotherapy either with a neoadjuvant or palliative intention.

## Conclusion

Chemotherapy is not yet effective in pancreatic ductal cancer. Thus, effects of chemotherapy on tumor tissue are normally not seen, unlike in other cancers, and no grading system can be applied. Acinar cell carcinomas develop by different pathways than ductal carcinomas. As shown in this case, neoadjuvant chemotherapy with 5-FU might well be a valuable step in the management of these patients.

## Consent

Written informed consent was obtained from the patient for publication of this case report and accompanying images. A copy of the written consent is available for review by the Editor-in-Chief of this journal.

## Competing interests

The authors declare that they have no competing interests.

## Authors' contributions

MD, FR, SK and RG performed surgery, follow up patient and helped in preparation of the manuscript. FD prepared the draft of the manuscript. CS prepared the pictures of the manuscript. Histopathological examinations were performed by DDD. All authors read and approved the final manuscript.
